# Report of a Case of Puerperal Diphtheria Involving Vagina and Endometrium1Read at the thirtieth annual meeting of the Medical Association of Central New York, at Buffalo, October 19, 1897.

**Published:** 1898-06

**Authors:** S. L. Elsner

**Affiliations:** Rochester. N. Y.


### REPORT OF A CASE OF PUERPERAL DIPHTHERIA INVOLVING VAGINA AND ENDOMETRIUM.[1]

By S. L. ELSNER, Rochester. N. Y.

PUERPERAL fever or puerperal sepsis is, at present, a very rare complication with which we, as skilled obstetricians, have to cope. It is still common enough to be called in by the midwife to make clean what she has carelessly allowed to become infected. Occasionally, however, we ourselves suffer by the gross negligence of a nurse whose training in asepticism has been overlooked. Aside from the interest that this case may have for us, I report it principally to impress upon you the importance of its prevention under like circumstances. After an investigation by the health board of the city of Rochester, they found that my nurse had cared for her own daughter with a sore throat only three days prior to her going into the service of my patient. It was further found that she called at a drug store and told the clerk that she wished him to prescribe for her daughter, since, if she called a physician, he might placard her house.

On the second morning after labor, when I called, I found that the bichloride douche ordered had not been given. The nurse excused herself by saying that the douche bag was not in perfect order, but that she would attend to it at once. On the fourth morning I found my patient with a slight elevation of temperature and on inquiring learned that the douche had not been given. Now, the ignorant attendant said that she did not give it because the patient flowed more freely, and she thought that this would be quite douche enough. I insisted upon the nurse's discharge, but she was retained because of her high proficiency in the culinary art. During the early morning of the fifth day this patient had her first chill and on my arrival I found her with a temperature of 104 3-5 degrees F.; pulse, 135; pain in the vagina, extending upward into the sides. Examination with speculum showed a marked change from a few days before. The entire vagina, cervix and as far as one could see into the cervical canal was coated with one mass of diphtheritic membrane. Dr. J. W. Whitbeck saw the case with me during the day and agreed that we had a diphtheritic infection to deal with. I at once made a culture and the specimen was loaded with the typical Klebs-Löffler bacillus. Subsequent cultures were likewise reported by the health board. On the sixth day she again had a chill with a temperature

1. Read at the thirtieth annual meeting of the Medical Association of Central New York, at Buffalo, October 19, 1897.

reaching 105 degrees. Her uterus had already been irrigated and the curette failed to find anything aside from diphtheritic membrane. This chill and fever was not ascribed entirely to the diphtheria, since some noticeable enlargement could be felt on the left side and diagnosticated as salpingitis. The local condition, however, was unaltered and she was given 2,000 units of antitoxin with the result of a gradual disappearance of the membrane in sixty hours. This brings us to the ninth day. The cultures now failed to show any Klebs-Löffler bacillus. The baby was also given a small dose of antitoxin and kept in the contagious pavilion of our city hospital by the side of the mother. Not the slightest symptoms of infection followed in the child.

The mother's treatment aside from the antitoxin was quinine internally and douches with intrauterine irrigation of lysol, 1 per cent. solution, locally. The vulva, after being shaved, was kept covered with an aseptic pad, and the labia separated by gauze. These dressings were changed every two or three hours. The case from this on presented nothing but the usual manifestations and symptoms of an ordinary pyosalpinx, with but one exception—the pulse would remain slow. The patient would have a chill, followed by a temperature of 103 to 105 degrees F., and on one occasion 106 1-5 degrees F., and yet the pulse-rate would not exceed 105. For the most of the time her pulse would run from 78 to 90. This slow heartbeat was afterward explained by the fact that when she reached a stationary normal temperature her pulse would vary between 56 and 68 per minute.

Between the ninth and sixteenth day of her illness she suffered considerable pain and her condition was precarious. Her temperature ranged from 103 to 106 degrees F., and she presented every appearance of a bad septic case. It was deemed advisable to attack the left tube and in order to decide as to the route, whether vaginal or abdominal section be made, I thought it advisable to thoroughly map out the local condition. After a rather severe vaginal examination, I noticed upon withdrawing my hand that it was besmeared with pus and blood. Upon inspecting the vagina the nurse found that it contained about half ounce of the same discharge. This manipulation ended all her symptoms. The next day her temperature was normal, her pain had vanished, the enlargement of the left tube could not be made out and the patient mended immediately and made a rapid recovery. She left the hospital on the twenty-sixth day after the birth of her child, just twenty-one days after the appearance of the diphtheritic membrane. She has remained well, and at present, about three months since her discharge, she presents no signs of her former trouble.

As a matter for statistics, I would state that this is the second case of puerperal vaginal diphtheria that I have seen, the first occurring seven years ago in a patient where I was able to trace the infection to a neighbor's house in which there were two cases of diphtheria. The nurse of this patient was taken suddenly ill, and, in a moment of thoughtlessness my patient's husband called upon the neighbor's wife to attend the patient during that day. This patient presented all the local symptoms in the vagina, but did not develop a salpingitis. She made a complete recovery in spite of the absence of antitoxin and a willing health board to rely upon for cultures.

83 North St. Paul Street.

